# Supplementary material for: Long-COVID-19 in Asymptomatic, Non-Hospitalized, and Hospitalized Populations: A Cross-Sectional Study
Source: J Clin Med. 2023 Mar 30;12(7):2613. doi: 10.3390/jcm12072613 (PMC10095523; doi:10.3390/jcm12072613)
Supplement: Supplementary file 1 [file jcm-12-02613-s001.zip › jcm-2235227-supplementary.pdf]

File S1. "Immune response to SARS-CoV-2 and post-COVID-19 in the upper respiratory tract".

SURVEY FORM

The aim of this study is to investigate the immune reaction against SARS-CoV-2 in the upper respiratory tract of people with COVID-19, to draw attention to the health problems they continue to experience after discharge, and to help illuminate the mechanism that causes these disorders.

Please answer the questions below and circle the number next to the option that is right for you. Thank you for your interest in our research.

Project manager: Assoc. Prof. Umut Gazi
Near East University Faculty of Medicine, Medical Microbiology and Clinical Microbiology

Participant name and surname: ....

Participant address: ....

Participant telephone numbers: (Home): .... (Cellular) ....

Date of survey: ..../ ..../............

Section I. Demographic information

1	Gender: **1.** Male **2.** Female
2	Age (Years): .....
3	Level of education: **1.** No education **2.** Primary school **3.** Secondary School **4.** High school **5.** Graduate **6.** Post-graduate
4	What is your occupation? .....
5	Marital status: **1.** Married **2.** Single
6	Smoking status: **1.** Yes **2.** No, I have never used **3.** I was using it, I quit
7	If you smoke, how long have been smoking? ….<br>How many cigarettes do you smoke per day? ....
8	If you have smoked and quit before, when did you quit?<br>How many cigarettes did you smoke per day?<br>How long have you smoked?
9	Do you have a diagnosed underlying disease? **1.** Yes **2.** No<br>If your answer is yes, please tick the appropriate one below (you can tick more than one option).

	□ Diabetes □ Hypertension □ Chronic heart disease □ Chronic respiratory disease<br>□ Chronic kidney disease □ Chronic liver disease □ Chronic neurological disease □ Cancer<br>□ Organ or bone marrow transplantation. □ Other (please specify): .....
10	When did you get COVID-19 disease?
11	Has any family member other than you had COVID-19 in the same home? **1.** Yes **2.** No
12	Have you been vaccinated against COVID-19? **1.** Yes **2.** No
13	If you have been vaccinated, which COVID-19 vaccine have you been vaccinated with?<br>**1.** Sinovac **2.** AstraZeneca **3.** Pfizer **4.** Johnson and Johnson **5.** Moderna
14	If you have been vaccinated, have you been vaccinated before or after you had the disease?<br>**1.** Before **2.** After
15	If you were vaccinated, when were you vaccinated?

Section II. Information about the COVID-19 disease

16	What were the first symptoms that bothered you the day you tested positive for COVID-19?<br>**1.** No symptom **2.** Fever **3.** Loss of appetite **4.** Chest pain **5.** Cough **6.** Diarrhea **7.** Dyspnea **8.** Sputum production. **9.** Fatigue **10.** Headache **11.** Haemoptysis **12.** Muscle pain **13.** Joint pain **14.** Nausea **15.** Sore throat **16.** Abdominal pain **17.** Runny nose **18.** Loss of taste **19.** Loss of smell<br>Other:
17	Have you been hospitalized for treatment? **1.** Yes **2.** No
18	If you were hospitalized, how long were you in the hospital?
19	Have you been hospitalized in the intensive care unit (ICU)? **1.** Yes **2.** No
20	If you were admitted to the ICU, how long did you stay in the ICU?
21	Was nasal oxygen therapy administered? **1.** Yes **2.** No
22	Was non-invasive ventilation applied? **1.** Yes **2.** No

23	Was invasive mechanical ventilation applied? **1.** Yes **2.** No
24	When were you discharged?

Section III. Information on health status after discharge from COVID-19

25	After discharge, how do you feel compared to your situation before you contracted COVID-19?<br><br>1. Normal 2. Worsening in health status (due to weakness, feeling tired) 3. Improvement in health status
26	Which of the following(s) increased in frequency after you were discharged?<br><br>**1.** Memory problems **2.** Dizziness **3.** Attention disorder **4.** Chest pain **5.** Palpitation **6.** Diarrhea **7.** Abdominal pain **8.** Nausea **9.** Vomiting **10.** Fatigue **11.** Dyspnea **12.** Cough **13.** Sore throat **14.** Muscle pain **15.** Joint pain **16.** Fever **17.** Headache **18.** Nasal obstruction **19.** Exuviation **20.** Hair loss **21.** Loss of appetite **22.** Sleep disorder<br><br>Other:
27	Which of the following have you experienced in the last week?<br><br>**1.** Memory problems 2. Dizziness 3. Attention disorder **4.** Chest pain **5.** Palpitation **6.** Diarrhea **7.** Abdominal pain **8.** Nausea **9.** Vomiting **10.** Fatigue **11.** Dyspnea **12.** Cough **13.** Sore throat **14.** Muscle pain **15.** Joint pain **16.** Fever **17.** Headache **18.** Nasal obstruction **19.** Exuviation **20.** Hair loss **21.** Loss of appetite **22.** Sleep disorder<br><br>Other:
28	What kind of change did you detect in your sense of taste after discharge?<br><br>**1.** No change **2.** Better than before illness **3.** Worse than before the illness
29	What kind of change did you detect in your sense of smell after discharge?<br><br>**1.** No change **2.** Better than before illness **3.** Worse than before the illness

Our survey is over. Thanks for your participation and contribution
